# Possible Role of Horizontal Gene Transfer in the Colonization of Sea Ice by Algae

**DOI:** 10.1371/journal.pone.0035968

**Published:** 2012-05-02

**Authors:** James A. Raymond, Hak Jun Kim

**Affiliations:** 1 School of Life Sciences, University of Nevada, Las Vegas, Nevada, United States of America; 2 Division of Polar Life Sciences, Korea Polar Research Institute, Incheon, South Korea; 3 Department of Polar Sciences, University of Science and Technology, Incheon, South Korea; J. Craig Venter Institute, United States of America

## Abstract

Diatoms and other algae not only survive, but thrive in sea ice. Among sea ice diatoms, all species examined so far produce ice-binding proteins (IBPs), whereas no such proteins are found in non-ice-associated diatoms, which strongly suggests that IBPs are essential for survival in ice. The restricted occurrence also raises the question of how the IBP genes were acquired. Proteins with similar sequences and ice-binding activities are produced by ice-associated bacteria, and so it has previously been speculated that the genes were acquired by horizontal transfer (HGT) from bacteria. Here we report several new IBP sequences from three types of ice algae, which together with previously determined sequences reveal a phylogeny that is completely incongruent with algal phylogeny, and that can be most easily explained by HGT. HGT is also supported by the finding that the closest matches to the algal IBP genes are all bacterial genes and that the algal IBP genes lack introns. We also describe a highly freeze-tolerant bacterium from the bottom layer of Antarctic sea ice that produces an IBP with 47% amino acid identity to a diatom IBP from the same layer, demonstrating at least an opportunity for gene transfer. Together, these results suggest that the success of diatoms and other algae in sea ice can be at least partly attributed to their acquisition of prokaryotic IBP genes.

## Introduction

In spring and summer, the underside of sea ice in many areas teems with algae, the great bulk of which are diatoms [1]. Over 200 species of sea ice diatoms have been identified [2]. A number of factors contribute to their success, including changes in photosynthesis and pigments to adapt to low light [3], [4], osmotic adjustments to tolerate high salinity [5], and changes in lipids to maintain membrane fluidity at low temperature (reviewed by [6]). In the case of the sea ice diatom *Chaetoceros*, hundreds of genes are involved in adapting to changes in light [7] and temperature [8].

Loss of extracellular water to freezing is another serious challenge to ice algae. In the case of diatoms, a unique characteristic is their secretion of ∼25 kDa ice-binding proteins (IBPs) into the surrounding environment. (Many proteins, such as fish antifreeze proteins, bind to ice; here, unless otherwise noted, IBP refers to proteins similar to those in diatoms). Although the extracellular concentrations are not high enough to significantly lower the freezing point, IBPs can improve the habitability of ice, such as by preventing freezing injury [9], [10], protecting and trapping pockets of water [11], [12], [13], and possibly serving to attach cells to ice. All eight species of sea ice diatoms examined so far produce IBPs or show IBP activity (Table S1), while all eight mesophilic diatoms examined so far do not show IBP activity (six species) [14] or have IBP-homologous genes in their genomes (two species) [15], [16]. In one diatom, there is also some evidence that IBP protein expression is increased by the cold, salty conditions in sea ice [17]. Together, these findings strongly suggest that IBPs are essential for survival of diatoms in sea ice and are one of the factors that have allowed them to so successfully colonize sea ice.

The apparent absence of IBP and IBP-homologous genes in the genomes of mesophilic diatoms raises the question of how the sea ice diatoms acquired the genes. Diatom-like IBPs with confirmed ice-binding activity have also been found in polar bacteria [18], [19] and cold-adapted fungi [20], [21], [22], and genes with full IBP domains are also found in several species of ice bacteria [18], leading to speculation that sea ice diatoms acquired their genes by horizontal transfer from bacteria [23], [24].

The recent accumulation of sequence data has shown that horizontal gene transfer (HGT) is a pervasive and powerful force in the microbial world [25], [26], including diatoms [16], [27]. Although most commonly observed between bacteria, HGT between prokaryotes and eukaryotes has been observed in both directions. In eukaryotes, newly acquired genes provide novelties that contribute to their adaptation to specific environments [28]. For example, acquisition of bacterial genes for glucuronidase by a fungus [29] and cutinase by an oomycete [30] have allowed them to exploit new food sources. Could an HGT event also help diatoms to expand their range into a new geographical region, such as the vast ice-covered oceans?

A major test of HGT is an incongruence between an accepted phylogeny of a group of organisms and the phylogeny of the gene of interest [31]. Previously, diatom IBP sequences were available for only four species, *Navicula glaciei* and *Fragilariopsis cylindrus* [23], *Fragilariopsis curta* [24] and *Chaetoceros neogracile* [32]. To obtain more robust trees, IBP sequences were obtained for three additional sea ice diatoms and two other Antarctic algae that were recently found to have diatom-like IBP genes. The latter include a prymnesiophyte, *Phaeocystis antarctica*, and a prasinophyte, *Pyramimonas gelidicola*, both of which occur near and within sea ice [33], [34]. We also report a similar IBP sequence from a bacterium that lives in the same layer of sea ice as the diatoms. Together, our results suggest that the acquisition of IBP genes by diatoms and other algae from prokaryotes was an essential factor in allowing them to expand their range to polar sea ice.

## Materials and Methods

All necessary permits for collections in Antarctica were obtained from the Office of Polar Programs of the U.S. National Science Foundation and the Korean Ministry of Foreign Affairs and Trade. None of the field studies involved endangered or protected species.

### Algal Cells

Axenic strains of the sea ice diatoms *Amphora* sp. (CCMP2378) and *Attheya* sp. (CCMP212) were obtained from the National Center for Marine Algae and Microbiota (NCMA, formerly CCMP), West Boothbay Harbor, ME, USA. Both species were originally collected from sea ice near Baffin Island, Canada. Based on their 18S rRNA sequences, *Attheya* sp. (JQ240486) was identified as *A. septentrionalis* (CCMP2084; acc. no. HQ912618; 100% 18S identity), which was also obtained from sea ice in Baffin Bay, and *Amphora* sp. (JQ240485) was closely related to *Amphora cf. capitellata* (AJ535158; 96.5% 18S identity) which was obtained in the Southern Ocean.

A sample of sea ice diatoms consisting of about 95% *Nitzschia stellata* was obtained from the platelet layer underlying the sea ice at McMurdo Sound, Antarctica in December 2002 and stored in RNAlater at -80^o^C. *N. stellata* was previously shown to have ice-binding activity [14]. For DNA analysis, a clump of ∼20 *N. stellata* cells was separated under the microscope from the original sample. *N. stellata* grows in a distinct branched pattern, simplifying their identification and separation from other cells.

Two Antarctic strains of the prymnesiophyte *Phaeocystis antarctica* (CCMP1374 and CCMP1871; both nonaxenic) were obtained from NCMA. They were originally collected near sea ice in McMurdo Sound and in Arthur Harbor, respectively.

*Pyramimonas gelidicola* AnM0046, a prasinophyte, was collected in seawater near King Sejong Station, King George Island, Antarctica. Cells were isolated with a Pasteur pipet under the light microscope and cultured. They were identified as *P. gelidicola* by their morphology under a scanning electron microscope and by their 18S rRNA sequence which was 99.9% identical to that of another Antarctic *P. gelidicola* strain (Acc. no. HQ111510).

### Bacterial Cells

Brown ice from the diatom layer of McMurdo Sound, Antarctica was retrieved from sea ice freshly broken by the icebreaker Polar Sea on 3 January 2003. The ice was allowed to melt. Diatoms sunk to the bottom and the clear supernatant was frozen, briefly centrifuged to expel brine, and then shipped to the University of Nevada, Las Vegas, where it was stored at -25^o^C until 2008. Samples of this ice were melted (approximate osmolality of the melt water was 200 mOsm kg^-1^) and plated on 2216 marine broth (Difco) agar plates, and incubated at 0^o^C. Many colonies were obtained within two weeks. The colonies were cultured in liquid 2216 medium at 10^o^C, centrifuged, and the supernatants were screened for ice-binding activity. Several colonies were found to produce ice-binding proteins. 16S rRNA sequencing of these colonies showed that they were virtually the same species.

### Ice-binding Activity

Ice-binding activity of an alga was determined by immersing an ice single crystal (a perfect crystal) in the supernatant (unconcentrated) of the culture medium at a temperature approximately 0.1^o^C below its freezing point and observing the degree of pitting that occurred on the ice basal plane [35].

### Sequencing of IBPs

At first, many attempts were made to obtain the *Attheya* sp. (CCMP212) and *Amphora* sp. (CCMP2378) IBP sequences by PCR using primers based on the *Navicula* and *Fragilariopsis* IBP sequences, but none were successful. In a second attempt, 3740 *Attheya* sp. ESTs were sequenced (GenBank acc. nos. JK725632 - JK729371) as described [13], but none matched an IBP sequence. Genomic sequencing was then tried.

Genomic DNA was prepared from *Amphora* sp. and *Attheya* sp. cultures with an Easy-DNA kit (Invitrogen) according to the manufacturer's instructions and sequenced on a Solid3 platform (Applied Biosystems) at the University of Oklahoma Health Science Center. Approximately 1 gigabase of 50-nt reads was obtained for each species (GenBank acc. nos. SRR026640 and SRR026631, respectively). For both species, the reads databases were queried with existing diatom IBP amino acid sequences using TBLASTN. Many primers were designed based on small contigs that resembled the 5′ and 3′ ends of IBP sequences. Of these, several produced PCR products of the expected size. The products were cloned and sequenced, yielding IBP-homologous sequences. For each species, blasting these sequences against the reads databases yielded 50-100 perfect 50-nt matches throughout the length of the query sequences, confirming that the PCR products and reads databases are consistent. In some cases, the 5′ and 3′ ends could be extended manually by finding overlapping reads.

The full 18S rRNA sequences of *Amphora* and *Attheya* (acc. nos. given above) were obtained by a combination of PCR and small reads assembly. The *Amphora* 18S sequence was found to contain two ∼120-nt introns.

*Attheya* IBP was purified by exploiting its ice-binding property and subjected to 2D electrophoresis as previously described [13]. Spots on the gel were subjected to MS/MS de novo sequencing at the University of California, Davis, as previously described [13].

The *Nitzschia stellata* IBP sequence was originally obtained from an aliquot of cells, about 5% of which were cells of other diatom species, that had been stored in RNAlater at -80^o^C. The cells were rinsed and lysed with lysis buffer [36]. A number of primers were made based on sequences of other IBPs. One primer pair: NIF 5′-gctgtcgacctcggcactgct-3′ from *Navicula glaciei* and F1R 5′-caatactaccagcaccgagtt-3′ from *Fragilariopsis cylindrus* yielded a 350-bp product that was homologous to the 5′ ends of other IBPs. Primer pairs consisting of a forward primer sequence from this sequence, F331 5′-taggcgacatggagaccgcattta-3′, and one of several reverse primers were then tried. A product of the expected size (380 bp) was obtained with a reverse primer R669C 5′-cggaatccaacgtgactgctgtc-3′ based on the IBP of *Chaetoceros neogracile* [32]. Using DNA prepared from isolated *N. stellata* cells (see above), the PCR was repeated with the same primers. The expected bands were obtained, although three rounds of PCR were needed to obtain the necessary amplification. The sequences of the bands were virtually identical to those obtained with the contaminated cells. The pure *N. stellata* DNA was also used to obtain a 1102-bp partial 18S rRNA sequence (acc. no. JQ240484), all of whose closest named matches were other *Nitzschia* species or a closely related species, *Bacillaria*.

A *Pyramimonas gelidicola* AnM0046 EST library was prepared and sequenced (W. Jung et al., ms. in preparation), and the sequences were submitted to GenBank. Two isoforms were assembled from the ESTs as follows: Isoform 1 (acc. nos. FS594586, FS594590, FS594790, FS594171, FS594432, FS594774), and isoform II (FS592765, FS594306). The 5′ end of isoform II was obtained by the chromosome walking method. The sequences were confirmed by sequencing genomic DNA.

The genome of *Phaeocystis antarctica* CCMP1374 was sequenced by G.M. Berg et al. (Stanford University) and submitted to NCBI in the form of 36-nt reads (ncbi.nlm.nih.gov/sra/SRP004354). The reads were downloaded and searched by local BLAST for sequences similar to existing IBP genes. Many hits were obtained and assembled into several short contigs corresponding to the 5′ and 3′ ends of IBP genes using BioEdit [37], from which several primers were designed. Genomic DNA was obtained by lysing CCMP1374 cells with lysis buffer. The obtained PCR products were sequenced and identified as IBP-like genes. Where necessary, the sequences were corrected by alignment with small reads. After submission of the paper, longer reads became available (see Discussion) and they largely confirmed the PCR-derived sequences.

*Flavobacterium frigoris* PS1 genomic DNA was prepared with a Wizard genomic DNA kit (Promega) according to the manufacturer's instructions. The DNA was sequenced using an Illumina GA IIX platform and assembled into 52 contigs by Chunlab (Seoul, Korea) (GenBank acc. no. PRJNA73123).

### Phylogenetic Trees

Phylogenetic trees were constructed with the neighbor-joining method using Mega5 [38]. Bootstrap values were obtained with 500 resamplings.

### Effect of IBP on Ice Structure

*Phaeocystis antarctica* CCMP1374 and *P. antarctica* CCMP1871 were cultured in 500 ml L1 seawater medium at 3.0^o^C for approximately 5 weeks, after which the cell-free culture media showed good pitting activity. The pitting activity was similar to that observed in water from brown ice [14], indicating that the IBP concentration was comparable to that in the natural environment. Approximately 65 ml of the culture media were centrifuged and the supernatants were transferred to new tubes. L1 medium was used as a control. The osmolalities of the three samples were adjusted to 980± 4 mOsm kg^-1^, corresponding to a freezing point of -1.82^o^C. The tubes were cooled to initiate freezing and then placed in a temperature bath at -3.0^o^C overnight. The next day the temperature was dropped to -4.0^o^C for 4 hr so that freezing occurred slowly. The tubes were then photographed.

## Results

### Distribution of IBPs

**Figure 1:**
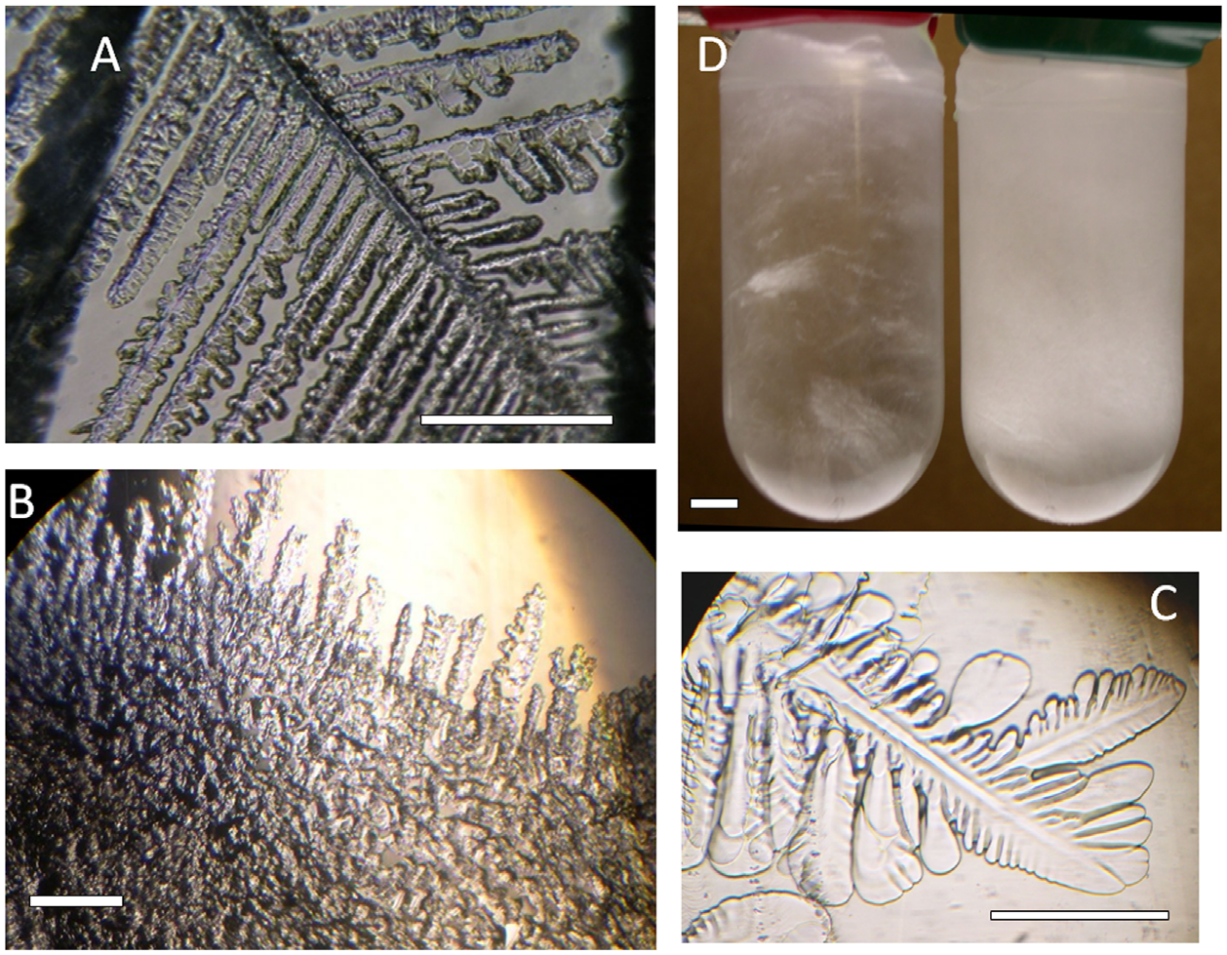
Ice-binding activities of *Pyramimonas gelidicola* AnM0046 and *Phaeocystis antarctica* CCMP1374. A, B, Distortion of growing ice by cell-free culture media of *P. gelidicola* and *P. antarctica*, respectively. Ice c-axis is normal to page. Scale bars, 1 mm. C, Growth of ice in seawater (control). Bar, 1 mm. D. Effect of *P. antarctica* IBP on the structure of sea ice. L1 seawater medium (left) and L1 medium containing a natural concentration of *P. antarctica* IBP (right) frozen at -4.0^o^C. The amount of ice in the two tubes is the same. Distortion of growing ice by the IBP greatly reduces the size of brine pockets. Bar, 1 cm.

Previous examinations of sea ice diatoms and non-sea ice diatoms suggested that IBP activity was confined to species living in sea ice. The extracellular media of 13 additional Antarctic diatoms were examined to test this hypothesis. These included six species of sea ice diatoms, two unidentified sea ice diatoms and five unidentified diatoms from ice-free tide pools (Table S1, all identified by "This study"). In addition to the diatoms, the prasinophyte *Pyramimonas gelidicola* AnM0046 and the prymnesiophyte *Phaeocystis antarctica* CCMP1374, both of which are found in and near sea ice, also showed strong extracellular ice pitting activity (Figure 1A, B) compared to a seawater control (Figure 1C).

### Effect of IBP on Structure of Ice

The fine structure imposed on growing ice by *P. antarctica* IBP (Figure 1B) can also be seen on a macroscale. Seawater ice made from culture medium of *P. antarctica* CCMP1374 cells containing a natural concentration of IBP (estimated at 1 µg ml^-1^, see below) was opaque and had a fine structure, while frozen unspent medium was semi-transparent and had a coarse structure (Figure 1D), indicating that the *P. antarctica* IBP caused the ice to form smaller brine pockets. Similar results were obtained for *P. antarctica* CCMP1871 (image not shown). Based on ice-pitting activity assays, the IBP concentration in the culture medium was similar to that in the natural environment [14].

### New IBP Sequences

A total of ten IBP DNA sequences were obtained for six microorganisms (three sea ice diatoms, two other sea ice-associated algae and a sea ice-associated bacterium) (Table S2). Other partial IBP sequences were found for *P. antarctica*, suggesting that it has multiple isoforms. Sequencing of genomic DNA showed that none of the sequences contained introns. All of the IBPs for which full sequences were obtained had N-terminal signal peptides. The sequences whose N-terminal sequences could not be determined (*Amphora, Nitzschia*, and *Phaeocystis*) are not necessarily secreted IBPs as they could be IBP domains in larger nonsecreted proteins. However, these species almost certainly have similar IBPs with N-terminal signal peptides because of their strong extracellular ice-binding activities. Like other IBPs, the sequences of the ten IBP genes are well conserved (Figure S1).

The *Attheya* IBP was ice-affinity-purified, revealing two isoforms on a 2D gel (Figure S2). De novo sequencing of the two spots yielded the peptide GAEFQGLLLVK in both spots, which closely matches part of the *Attheya* sequence, GAEMQGILLVK. The molecular weight and pI of the spots (27 kDa, pI 4.3) are close to the values predicted from the full sequence. These results confirm that the sequenced gene encodes an ice-binding protein.

### Phylogenetic Relationships

**Figure 2:**
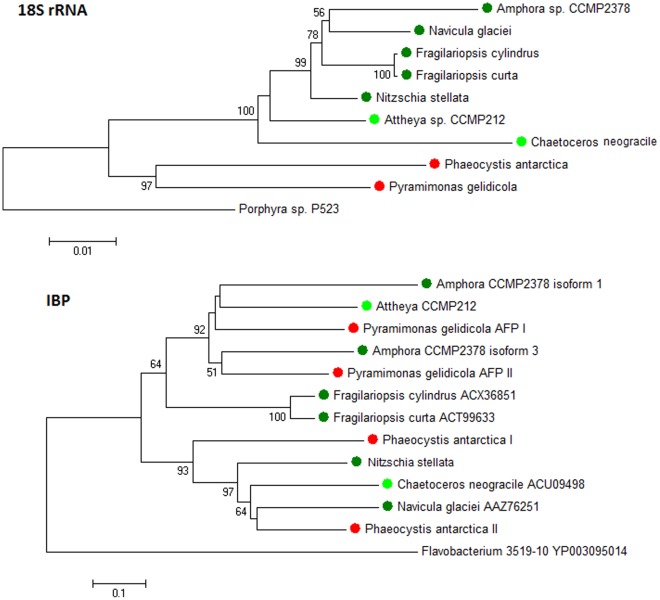
Incongruence between phylogenies of polar algae based on 18S rRNA and ice-binding protein sequences trees. Dark green symbols, Pennate diatoms; light green symbols, centric and quasi-centric diatoms; red symbols, prymnesiophyte and prasinophyte algae. IBP tree rooted with a bacterial IBP. Bootstrap values <50 are not shown. Accession numbers for the 18S sequences are *Amphora* sp., JQ240485; *Navicula glaciei*, EF106788; *Fragilariopsis cylindrus*, AY485467; *Fragilariopsis curta*, EF140623; *Nitzschia stellata*, JQ240484; *Attheya* sp., JQ240486; *Chaetoceros neogracile*, EU090012; *Phaeocystis antarctica*, X77477; *Pyramimonas gelidicola*, EU141942, *Porphyra* sp. P523, GU319853. Accession numbers of IBPs not given in the figure are given in Table S2.

Figure 2 compares the Neighbor-joining phylogenies of the algal IBP amino acid sequences and the 18S rRNA sequences of the same species. The 18S tree, rooted on the 18S sequence of the more primitive red alga *Porphyra*, shows the expected groupings, with diatoms forming a cluster separate from *Phaeocystis* (a prymnesiophyte) and *Pyramimonas* (a prasinophyte). *Chaetoceros*, a centric diatom, also is separated from the pennate diatoms and from *Attheya*, which appears to have an intermediate position between centric and pennate diatoms [39]. The IBP tree bears no resemblance to the 18S tree: the centric diatom *Chaetoceros* clusters with some pennate diatoms and *Phaeocystis*, while other pennates (*Fragilariopsis* and *Amphora*) form a separate group with the intermediate *Attheya* and *Pyramimonas*. Similar IBP trees were constructed with the maximum likelihood and maximum parsimony methods. Such incongruities are generally taken as evidence for HGT. An alternate explanation involving gene duplication and gene loss seems unlikely, as discussed below. Moreover, the existence of three clusters with high bootstrap values (>90%) raises the possibility of separate gene transfer events.

**Figure 3:**
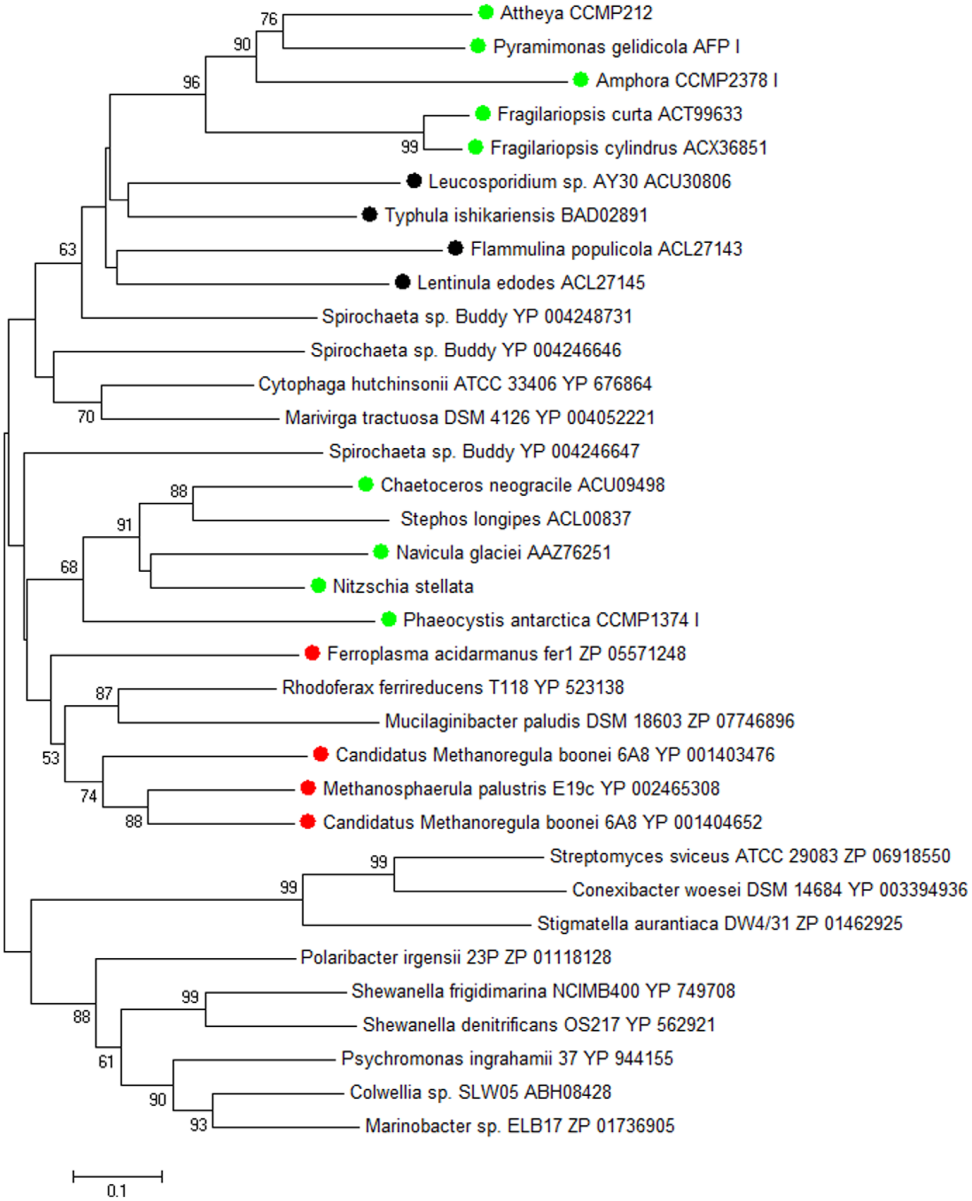
Neighbor-joining tree based on amino acid sequences showing best matches of algal (green symbols) and fungal (black symbols) IBPs. None of the matches except the IBP of the bacterium *Colwellia* SLW05 have been confirmed to be bona fide IBPs. The tree includes all genes in GenBank that match any of the algal IBPs to within an expect value of 1xe^-25^. Other than self matching sequences, all nearest matches are of bacterial (unmarked) and archaeal (red symbols) proteins. Numbers at nodes indicate bootstrap values obtained with 500 resamplings. Bootstrap values <50 are not shown. *Stephos* is a copepod whose IBP appears to have been acquired from *Chaetoceros*. Accession nos. of IBPs are shown in Table S2.

Additional evidence of HGT is the finding that all of the proteins in the database that most closely match the algal IBPs (to within an expect value of 1e^-25^), apart from other known algal and fungal IBPs, are prokaryotic genes (Figure 3). Finally, all the new IBP sequences, like those of *N. glaciei*, *F. cylindrus* and *C. neogracile* (see Discussion), lack introns.

### Sea Ice Bacteria as a Potential Source of IBP Genes

**Figure 4:**
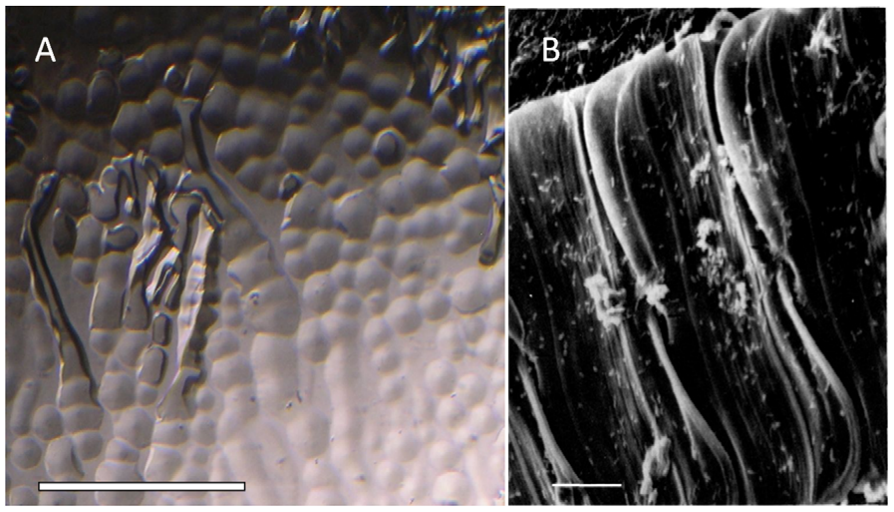
Bacteria in the diatom layer of sea ice. (A) Basal plane of an ice single crystal grown in the presence of culture medium of *F. frigoris*. An ice-binding protein in the culture medium binds to the pit faces, allowing growth in the c-axis direction (normal to basal plane). Scale bar, 1 mm. (B) Epiphytic bacteria on a chain of the sea ice diatom *Amphiprora* sp. (not the *Amphora* of this study). Reproduced from Sullivan *et al*. [46] with permission. Scale bar, 2 µm.

To see if IBP+bacteria were present in the diatom layer of sea ice, an attempt was made to recover bacteria from a sample of ice from the diatom layer that had been stored for 5 yrs at -25^o^C. Many colonies were obtained and three of 12 colonies examined were found to secrete ice-active proteins (Figure 4A). The 16S rRNA sequences of the three colonies were virtually the same, indicating a single active species. The 16S sequence (GenBank acc. no. JQ712371) was >99% identical to those of several flavobacteria isolated from Arctic and Antarctic marine sediments and >98% identical to several strains from Arctic sea ice. The strain's closest named match (99.2% identical) was the Arctic marine bacterium *Flavobacterium frigoris* KOPRI_22249, and was thus designated as *F. frigoris* strain PS1 (after the icebreaker Polar Sea). Sequencing of its genome revealed one typical IBP gene (acc. no. JQ712369) encoding a 26.1 kDa protein with an N-terminal signal peptide, and another gene (acc. no. JQ712371) encoding a large (∼240 kDa) hypothetical protein with an IBP-like domain and an N-terminal signal peptide. The former protein with a signal peptide is most likely the source of the extracellular ice-binding activity of *F. frigoris* PS1. The sequence most closely matched a predicted IBP from *Polaribacter irgensii* 23-P (60% a.a. identity, 72% similarity) and the IBP of *Colwellia* SLW05 (58% identity, 74% similarity). Both of these species are found in sea ice but only *F. frigoris* PS1 was obtained from the diatom layer. Among algal IBPs, the bacterial sequence most closely matched the IBP of *Nitzschia stellata* (47% identity, 66% similarity). This result, while not sufficient to prove HGT, confirms that at least one IBP-producing bacterium is found in the same microhabitat as sea ice diatoms, and demonstrates that an opportunity for the exchange of genes exists.

## Discussion

Here we report several new IBP genes from microorganisms associated with sea ice. The fact that these genes occur in a wide range of ice-associated microorganisms, including bacteria, several types of algae and fungi (see Introduction for references; also this study), and even a sea ice copepod (see below) leaves little doubt about their usefulness in icy environments. The products of the genes are active, as shown by the ice affinity of the *Navicula* [23] and *Attheya* (this study) IBPs, the ice-pitting activity of a recombinant protein [17] and the fact that all the new microbial species examined in vitro in this study showed ice-pitting activity.

Recently, G.M Berg et al. (Stanford University) deposited a large number of long (∼900 bp) reads of the genomic sequence of *Phaeocystis antarctica* in NCBI <http://www.ncbi.nlm.nih.gov/Traces/trace.cgi?&cmd=retrieve&val=species_code%3D%22PHAEOCYSTIS%20ANTARCTICA%22&retrieve=Submit>,and more than 200 of these reads match IBP and IBP-like genes. Most of the reads matching our isoform 2 encode N-terminal signal peptides and thus probably encode true, secreted IBPs, while the reads matching our isoform 1 appear to encode larger proteins with a C-terminal IBP domain. Although the functions of the latter are unclear, one possibility is that the IBP domains are on the cell surface where they help the cells to bind to ice.

The function of the secreted IBPs, although not known with certainty, may be to preserve a liquid environment which is essential for survival in ice. The fine structure of sea ice containing *P. antarctica* IBP (Figure 1C) is a consequence of its highly distorted growth (Figure 1B), leading to the formation of small brine pockets. *Phaeocystis antarctica* has been shown to have an unusually high freeze-thaw tolerance (Tang 2010), possibly because of this fine structure. The IBP concentration in the medium is not known, but based on the pitting activity of a recombinant IBP from the diatom *Fragilariopsis cylindrus* [17], it is probably near 1 µg ml^-1^. The IBP of another ice-associated alga, *Chlamydomonas* sp. CCMP681, although unrelated to the diatom IBPs, has a similar effect on the structure of sea ice, and was shown to retard the drainage of brine [13]. Similarly, extracellular polymeric substances, possibly a glycoprotein, produced by a sea ice diatom, *Melosira arctica*, created convoluted ice-pore morphologies in sea ice, potentially increasing its habitability and primary productivity [11]. The recent finding that bacterial genes are the closest matches to many diatom genes indicate that HGT between bacteria and diatoms is pervasive and much higher than in other sequenced eukaryotes [16]. The large number of foreign genes has helped to make diatoms the most successful group of eukaryotic phytoplankton in the modern ocean [27]. It is clear that their success extends to sea ice where they are an important component of the Southern Ocean ecosystem [1]. The present data strongly suggest that this success is at least partly due to the acquisition of IBP genes.

Potential mechanisms by which foreign DNA could be incorporated into eukaryotic cells include phagotrophy and viral transduction [28]. Among autotrophic algae, large DNA viruses may be an important transfer agent between eukaryotic cells, as they have been shown to massively infect algal blooms as well as to carry eukaryotic host genes [40], [41]. How bacterial genes could be incorporated in these viruses is unclear. One possibility is that sea ice itself could provide a suitable environment: sea ice has high concentrations of extracellular DNA and viral DNA compared to the concentrations in the underlying seawater [42]–[43], and together with its numerous surfaces for attachment, could be a hotspot for HGT in the marine environment [42].

A major argument for the horizontal transfer of IBP genes from bacteria to diatoms is the striking incongruence between the 18S rRNA and IBP trees (Figure 2), indicating a polyphyletic origin of the IBP genes. The mismatch is amplified by the seemingly random positions of the non-diatoms *Phaeocystis* and *Pyramimonas* in the trees. It should be noted that the incongruence in the trees does not rule out the possibility of gene duplication and differential gene loss as an alternate explanation [31]. However, the distribution of IBPs among polar algae (Figure 2) would appear to require the existence of multiple genes in an early ancestor followed by multiple gene losses, a less parsimonious explanation than HGT.

The finding that all the closest matches of the algal IBP genes are prokaryotic genes (apart from other known algal and fungal IBP genes) provides further evidence of HGT. Unexpected topologies such as this, in which eukaryotic genes cluster with prokaryotic genes, has been increasingly used to identify HGT [44], including the bacterial origin of hundreds of genes in the genomes of the mesophilic diatoms *Phaeodactylum tricornutum* and *Thalassiosira pseudonana*, [16].

Further evidence of a prokaryotic origin of the new algal IBP genes is that each of the new algal IBP sequences lack introns. In addition, a preliminary search of over 50 *P. antarctica* reads with complete or nearly complete IBP domains also revealed no introns. Introns are also absent in the three other known diatom IBP genes: *N. glaciei* (J.R., unpublished data), *F. cylindrus* [17] (GenBank acc. nos. GQ232744-GQ232750) and *C. neogracile* (E. Jin, Hanyang University, personal communication). In contrast, in the genomes of diatoms *P. tricornutum* and *T. pseudonana*, other genes have an average of approximately 1 intron [16]. However, each of the fungal IBPs that has been examined has several introns [21], [22], possibly indicating an earlier acquisition of IBP genes by fungi. The unrelated *Chlamydomonas* IBP mentioned above also has several introns; it is unique in the databases and its origin remains obscure [13].

As the *Phaeocystis* and *Pyramimonas* IBP genes indicate, IBP genes have spread to other polar algae besides diatoms. Surprisingly, an IBP gene has also been found in a sea ice copepod that feeds on diatoms [45]. The copepod IBP, which is 66% identical to the *Chaetoceros* IBP at the amino acid level, was found to be expressed in every cell type examined, including oocytes, ruling out the possibility of an endosymbiont or contamination from gut contents.

Antarctic sea ice, especially the bottom layer where diatoms are found, teems with bacteria, approaching 10^6^ cells ml^-1^ [46], with epiphytic bacteria accounting for up to 30% of the total bacterial biomass [47]. In the case of the diatom *Amphiprora* (not the *Amphora* of this study), whose cells are densely colonized with bacteria in summer (Figure 3B), there may be a mutualistic interaction [47]. Such intimate contact appears to provide ample opportunity for HGT, and the identification of *F. frigoris* in this layer indicates the presence of at least one potential IBP gene donor. Although no other bacteria from this layer have been screened for IBP genes or IBP activity, other potential donors are most likely present. One is *Polaribacter irgensii* P-23, whose genome contains a putative 27.5 kDa IBP gene with a probable signal peptide (acc. no. ZP_01118128). Although this strain was obtained from the water column, another strain of *P. irgensii* was obtained from the bottom of an ice core in Antarctic sea ice [48]. Another potential donor is *Colwellia* SLW05, which is a known IBP-producing species [19]. This strain was obtained from a melt puddle on the sea ice surface, but a strain with a virtually identical 16S sequence (acc. no. DQ060396) was obtained from the interior of Arctic sea ice [49].

The presence of IBP+bacteria in sea ice does not necessarily mean that they are the source of diatom IBP genes. Diatoms may have acquired IBP genes from bacteria in other cold habitats or even mesophilic habitats, which could have allowed them to move into colder regions. Presently, the bacterial gene that most closely matches a diatom IBP is from *Rhodoferax ferrireducens*, a psychrophilic species isolated from marine sediments in Virginia, USA [50]. The gene (acc. no. YP_523138) encodes a hypothetical protein of 51.7 kDa that contains a complete IBP domain, whose amino acid sequence is 58% identical to that of the *Navicula* IBP. In addition, over a 569-bp sequence, the nucleotide sequences are 65% identical. The function of the bacterial gene is unknown, but its IBP domain might serve to bind to a substrate other than ice. It should be noted that many bacterial strains with16S rRNA sequences nearly identical (>97.5%) to that of *R. ferrireducens* have been isolated from icy habitats, including Arctic sea ice (Table S3.). However, reads matching the *R. ferrireducens* IBP-like gene or 16S rRNA were not found in metagenomic datasets from Antarctic sea ice and seawater (a total of 2.3 Gb) submitted to the NCBI small reads archive by J. Deming et al. (University of Washington).In addition to its IBP activity, *F. frigoris* PS1 is remarkably freeze-tolerant, as shown by its ability to recover after 5 yrs at -25^o^C without an added cryopreservative (this study). Contributing factors could have been the bacterium's own IBP as well as diatom IBPs which were abundant in the ice in which it was stored. The *F. frigoris* genome, with an estimated size of 3,934,101 bases, encodes 3584 proteins, 37 of which most closely matched those from three other species of sea ice bacteria (*Polaribacter irgensii*, *Psychroflexus torquis* and *Psychromonas ingrahamii*) and 18 of which most closely matched genes from an IBP-producing and remarkably freeze-tolerant bacterium recovered from deep in the Vostok ice core [18], which suggests that *F. frigoris* PS1 is well adapted to surviving in ice.

Together, our results strongly suggest that the IBP genes of sea ice diatoms were acquired from bacteria, possibly in separate events, although not necessarily in sea ice, and that the acquisition of these genes was an essential factor in allowing the diatoms to expand their range to polar sea ice.

## Supporting Information

Figure S1 **Alignment of IBPs sequenced in this study.** *Amphora* sp. III is nearly identical to Amphora sp. II and is not shown.
(DOCX)

Figure S2 **Two-dimensional gel showing two isoforms of Attheya sp. CCMP212 IBPs purified by ice affinity.** Vertical scale is molecular weight in kDa and horizontal scale is pI.
(TIF)

Table S1 **Polar diatoms with and without ice-binding activity.**
(DOCX)

Table S2 **Characteristics of IBPs sequenced in this study.**
(DOCX)

Table S3 **Representative bacterial isolates from icy habitats with 16S rRNA sequences similar to that of** ***Rhodoferax*** **ferrireducens.**
(DOCX)
