# Supplementary figures and images for: Possible Role of Horizontal Gene Transfer in the Colonization of Sea Ice by Algae
Source: PLoS One. 2012 May 2;7(5):e35968. doi: 10.1371/journal.pone.0035968 (PMC3342323; doi:10.1371/journal.pone.0035968)

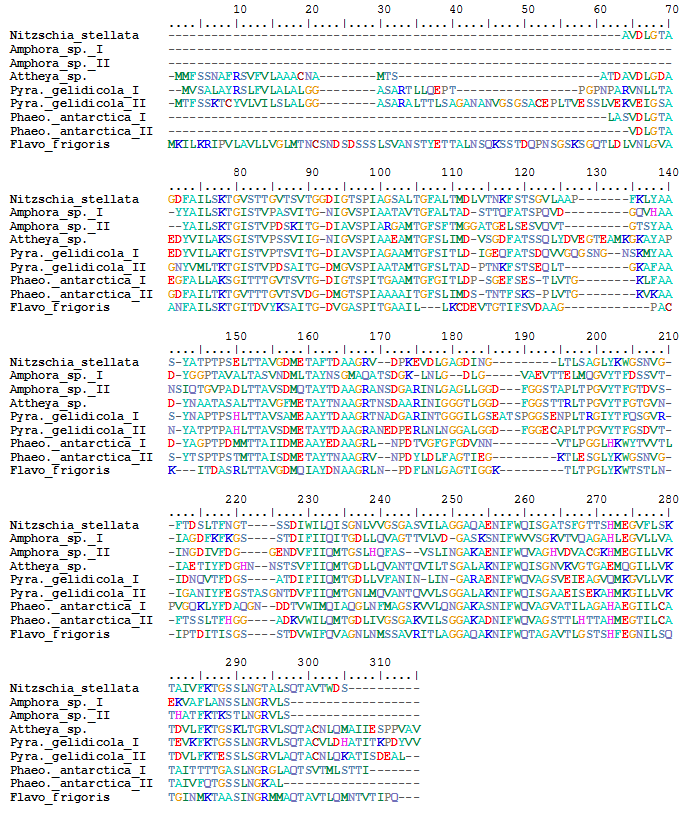

Supplement: Figure S1 — Alignment of IBPs sequenced in this study. Amphora sp. III is nearly identical to Amphora sp. II and is not shown. (DOCX) [file pone.0035968.s001.docx]

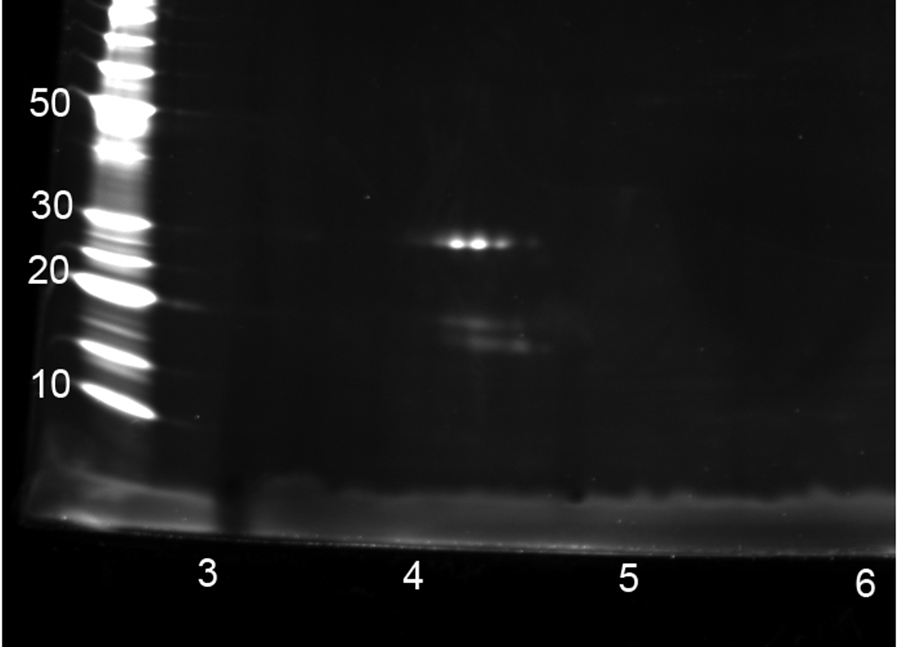

Supplement: Figure S2 — Two-dimensional gel showing two isoforms of Attheya sp. CCMP212 IBPs purified by ice affinity. Vertical scale is molecular weight in kDa and horizontal scale is pI. (TIF) [file pone.0035968.s002.tif]
