# Supplementary material for: Possible Role of Horizontal Gene Transfer in the Colonization of Sea Ice by Algae
Source: PLoS One. 2012 May 2;7(5):e35968. doi: 10.1371/journal.pone.0035968 (PMC3342323; doi:10.1371/journal.pone.0035968)
Supplement: Table S1 — Polar diatoms with and without ice-binding activity. (DOCX) [file pone.0035968.s003.docx]

Table S1. Polar diatoms with and without ice-binding activity.

| Habitat | Species | Ice-  active? | Ref. |
| --- | --- | --- | --- |
| Ant-arctic sea ice | *Nitzschia stellata* | y | [14] |
|  | *Porosira pseudodenticulata* | y | [14] |
|  | *Berkeleya sp.* | y | [51] |
|  | *Amphiprora kufferathii* | y | [51] |
|  | *Pleurosigma sp.* | y | [51] |
|  | *Navicula glaciei* | y | [23] |
|  | *Chaetoceros neogracile* | y | [32] |
|  | *Fragilariopsis cylindrus* | y | [17] |
|  | *Nitzschia curta CCMP 553* | y | This study |
|  | *Unidentified CCMP2748* | y | This study |
|  | *Unidentified CCMP2749* | y | This study |
| Arctic sea ice | *Porosira glacialis CCMP671* | y | This study |
|  | *Amphora sp. CCMP2378* | y | This study |
|  | *Pauliella taeniata CCMP1115* | y | This study |
|  | *Synedra hyperborea CCMP1422* | y | This study |
|  | *Attheya sp. CCMP212* | y | This study |
| Ant-arctic ice-free tide pools | *Unidentified CCMP2321* | n | This study |
|  | *Unidentified CCMP2323* | n | This study |
|  | *Unidentified CCMP2325* | n | This study |
|  | *Unidentified CCMP2326* | n | This study |
|  | *Unidentified CCMP2327* | n | This study |
