# Supplementary material for: Possible Role of Horizontal Gene Transfer in the Colonization of Sea Ice by Algae
Source: PLoS One. 2012 May 2;7(5):e35968. doi: 10.1371/journal.pone.0035968 (PMC3342323; doi:10.1371/journal.pone.0035968)
Supplement: Table S2 — Characteristics of IBPs sequenced in this study. (DOCX) [file pone.0035968.s004.docx]

Table S2. Characteristics of IBPs sequenced in this study.

| Species, location | Iso- form | Cover-  Age | No. a.a. residues | Signal peptide? | Acc. No. |
| --- | --- | --- | --- | --- | --- |
| *Attheya* sp. CCMP212  Canadian Arctic sea ice |  | full | 268 | yes | JQ240481 |
| *Amphora* sp. CCMP2378  Canadian Arctic sea ice | I | partial | 199 | nd | JQ240478 |
|  | II | partial | 204 | nd | JQ240479 |
|  | III | partial | 204 | nd | JQ240480 |
| *Nitzschia stellata*  McMurdo Sound sea ice |  | partial | 217 | nd | JQ240477 |
| *Phaeocystis antarctica* CCMP1374  McMurdo Sound Antarctica water column | I | partial | 222 | nd | JQ240482 |
|  | II | partial | 203 | nd | JQ240483 |
| *Pyramimonas gelidicola* AnM0046  water column near King George Island, Antarctica | I | full | 275 | yes | JQ743499 |
|  | II | full | 287 | yes | JQ743500 |
| *Flavobacterium frigoris* PS1  McMurdo Sound Antarctica sea ice |  | full | 276 | yes | PRJNA73123 |

nd, not determined
