# Supplementary material for: Possible Role of Horizontal Gene Transfer in the Colonization of Sea Ice by Algae
Source: PLoS One. 2012 May 2;7(5):e35968. doi: 10.1371/journal.pone.0035968 (PMC3342323; doi:10.1371/journal.pone.0035968)
Supplement: Table S3 — Representative bacterial isolates from icy habitats with 16S rRNA sequences similar to that of Rhodoferax ferrireducens. (DOCX) [file pone.0035968.s005.docx]

Table S3. Representative bacterial isolates from icy habitats with 16S rRNA sequences similar to that of *Rhodoferax ferrireducens*.

| Location | Acc. No. | % ID |
| --- | --- | --- |
| pack ice, Arctic Ocean | AF468446 | 99.1 |
| permafrost, Spitsbergen | EF451697 | 98.9 |
| Arctic stream, Alaska | FJ849070 | 98.8 |
| iron-rich snow, Japan | AB504935 | 98.8 |
| snow, Ellesmere Island | HQ327175 | 98.6 |
| beneath Arctic glacier, Canada | DQ628928 | 98.6 |
| lake, Transantarctic Mountains | FR691423 | 98.6 |
| Collins glacier, Antarctica | EU636030 | 98.5 |
| glacier, New Zealand | AY315172 | 98.4 |
| Lake Fryxell, Antarctica | AY609198 | 98.4 |
| Taylor Glacier, Antarctica | DQ677854 | 98.4 |
| glacier meltwater, Spitsbergen | FM955857 | 98.4 |
| lake mats, Antarctica | AJ440986 | 98.3 |
| high elevation snow, Switzerland | AJ867754 | 98.3 |
| penguin droppings, Antarctica | AY218607 | 98.2 |
| Puruogangri glacier, Tibet | DQ418523 | 98.0 |
| Lake Vida ice cover, Antarctica | DQ521494 | 97.9 |
| red snow, Antarctica | AB519660 | 97.8 |
| Lake Hoare, Antarctica | DQ535024 | 97.7 |
| fresh water lake, Antarctica | AB630460 | 97.5 |
